# Toothache

**Published:** 1893-09

**Authors:** 


### Toothache.

Dr. Gatchkovsky has discovered that the "electric breeze" is an admirable calmative agent in odontalgia. The brush is placed over the aching tooth and the patient placed upon an insulated chair, as in the administration of static electricity. Curiously enough, he has found this current to exercise a sedative effect not only upon nervous toothache, but also those of inflammatory origin and due to a pulpitis or periostitis. The pain begins to diminish in two or three, and to disappear completely within five or six minutes. The pain may return, but it will be less intense and yield to a few sittings of at least ten minutes duration. Under the influence of this current the gums become manifestly pale at the time that the toothache disappears. He has employed it in seventy-five cases, with but three failures.—*La Semaine Med.*

